# Evaluation of Flexural Toughness of Concrete Reinforced with High-Performance Steel Fiber

**DOI:** 10.3390/ma16206623

**Published:** 2023-10-10

**Authors:** Do-Hyuck Koo, Jong-Sun Kim, Sun-Hee Kim, Sang-Wook Suh

**Affiliations:** Department of Architectural Engineering, Gachon University, Seongnam-si 13120, Republic of Korea; dhkoo@nmc.or.kr (D.-H.K.); kjswno1@gachon.ac.kr (J.-S.K.); shkim6145@gachon.ac.kr (S.-H.K.)

**Keywords:** flexural test, residual stress, arched steel fiber, high-performance, concrete

## Abstract

In this study, a flexural test and residual stress evaluation using the aspect ratio (65 and 80) and steel fiber content (20, 30, and 40 kg/m^3^) as variables were conducted according to the EN 14651 standard to investigate the flexural toughness of concrete reinforced with high-performance arched steel fibers. The result of the flexural test show that the residual stress was 114.5% higher in the test specimen with high curvature and high content of arched steel fibers than that in the other conditions. In addition, the energy absorption capacity of arched steel fiber-reinforced concrete increased by 138.88% compared to concrete.

## 1. Introduction

Concrete has low tensile or flexural strength. Moreover, it is brittle and has low crack resistance due to its low energy absorption capacity. However, steel fiber-reinforced concrete (SFRC) is a material that improves flexural strength, tensile strength, shear stress, and brittle failure, as well as the shear strength and binding force of concrete. Accordingly, active research is being conducted worldwide to replace shear and confinement reinforcement with steel fibers [1,2].

Steel fibers currently used in concrete include hooked and straight types, which differ in properties such as length, diameter, and tensile strength. Factors affecting the properties of SFRC include the properties of the fibers and concrete and the specimen size [1,2].

In ACI 544.4R [3], the force or strength imparted by fibers in the cracked concrete cross-section is considered the residual strength and residual stress of fiber-reinforced concrete. The area of the load–displacement curve of fiber-reinforced concrete is known as the energy absorption by the concrete, i.e., toughness. For high-strength concrete (60–80 MPa), steel fibers with high tensile strength increase the residual stress. With increasing fiber aspect ratio, energy dissipation capability improved significantly, and the residual stress increased without stress reduction [4,5]. In the case of 30 MPa-class hooked SFRC, the residual flexural strength in the serviceability limit state and ultimate limit state increased with increasing fiber content and aspect ratio, with significant improvement in energy dissipation capability according to the results of fracture energy comparison [6]. According to Venkateshwaren et al. [7], replacing single-hooked steel fibers with double-hooked steel fibers increased the residual stress by up to 12%. Tiberti et al. [8] reported that the residual stress increased with increasing aspect ratio and fiber content in SFRC, but the workability of concrete decreased. In addition, the fiber content was found to be less effective above a certain level.

Sakthivel and Aravind conducted experiments to confirm the effect of the steel fiber type (hooked or crimped) on the cracking, yield, peak, and failure loads in RC beams. Consequently, the total energy absorption capacity of the RC beam showed that the incorporation of crimped fibers was higher than that of hooked fibers [9]. Chen et al. conducted flexural tensile tests to determine the effects of novel multiple hooked-end steel fibers with various strength grades. As the amount of fiber mixed increased, the behavior after cracking improved [10]. Wang et al. [11] experimentally confirmed the effect of steel fibers and concrete strength on the flexural toughness of ultra-high-performance concrete. The best flexural toughness efficiency was obtained at a fiber mixing ratio of 2%. Unlike steel materials, standardizing the properties of SFRC is challenging because they vary depending on variables such as the aspect ratio and steel fiber content.

ACI 318 [12] specifies the performance of SFRC using the flexural toughness index and steel fiber content—the most important properties of SFRC. As the mechanical properties of SFRC are mainly determined by the steel fiber content and aspect ratio, a flexural test considering the type, aspect ratio, and content of steel fibers is required to be used.

Therefore, this study evaluated the results regarding the suitability and reliability of arched SFRC with respect to those in previous studies based on arched and hooked steel fibers. The study considered the geometry, tensile strength, and content of fibers (among the properties that affect SFRC) as variables. For the comparison with arched steel fibers, the geometry of the steel fibers used in previous studies was limited to single-hooked steel fibers. In addition, the flexural performance and energy absorption capacity of SFRC were evaluated.

## 2. Flexural Test on Arched SFRC

### 2.1. Overview

This study aimed to investigate the effects of using arched steel fibers on the flexural properties of concrete. Table 1 shows the mix proportions of the 35 MPa concrete used in this study. The water-to-binder (W/B) ratio, the fine aggregate ratio (S/a), and the maximum size of coarse aggregate were set to 36.4%, 55.1%, and 25 mm, respectively. The size of the coarse aggregate for general structures was set to 25 mm, according to concrete specifications in Korea.

According to Kim et al. [6], residual flexural strength and energy dissipation capability improved according to the fiber content and aspect ratio. Therefore, in this study, the content of steel fibers (20, 30, and 40 kg/m^3^) and their aspect ratio (65 and 80) were used as variables to evaluate the flexural properties of concrete according to the content, aspect ratio, and tensile strength of arched steel fibers as shown in Table 2. Four specimens were prepared for each variable, resulting in 48 specimens.

**Figure 1:**
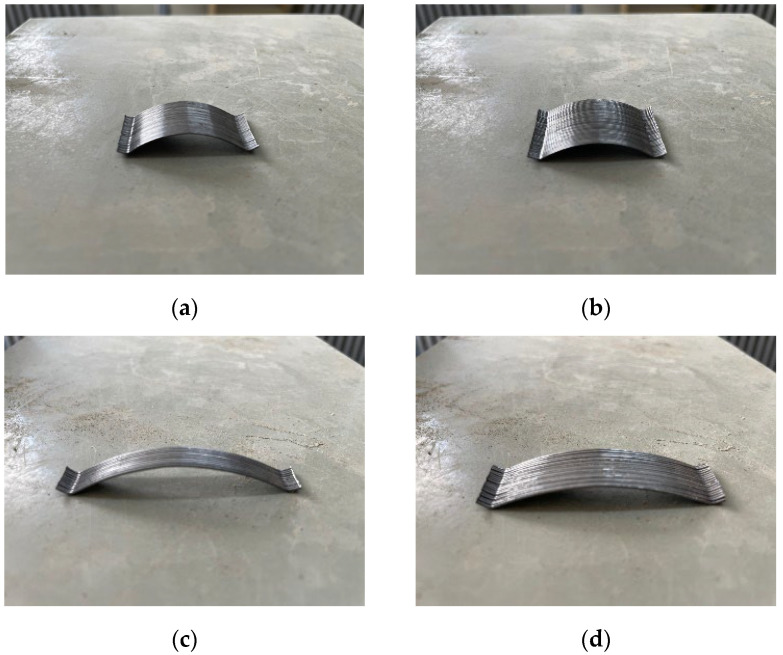
Shape of the arched steel fibers: (**a**) 65 R20; (**b**) 65 R27; (**c**) 80 R40; (**d**) 80 R51.

The arched steel fibers produced by the steel company Kosteel, Seoul, in Korea, as shown in Figure 1, were used in this study. The company produces approximately 400,000 tons of mild steel wire, rebar, and processed products annually. For arched steel fibers, a flexural toughness test was performed on commercially available SUPER BUNDREX^®^, Kosteel, Seoul, in Korea. The steel fibers shown in Figure 1a,b have an aspect ratio of 65, a length of 35 mm, a diameter of 1.55 mm, and a tensile strength of 1250 MPa, while the fibers shown in Figure 1c,d has an aspect ratio of 80, a length of 60 mm, a diameter of 0.75 mm, and a tensile strength of 1100 MPa.

### 2.2. Experimental Method

**Figure 2:**
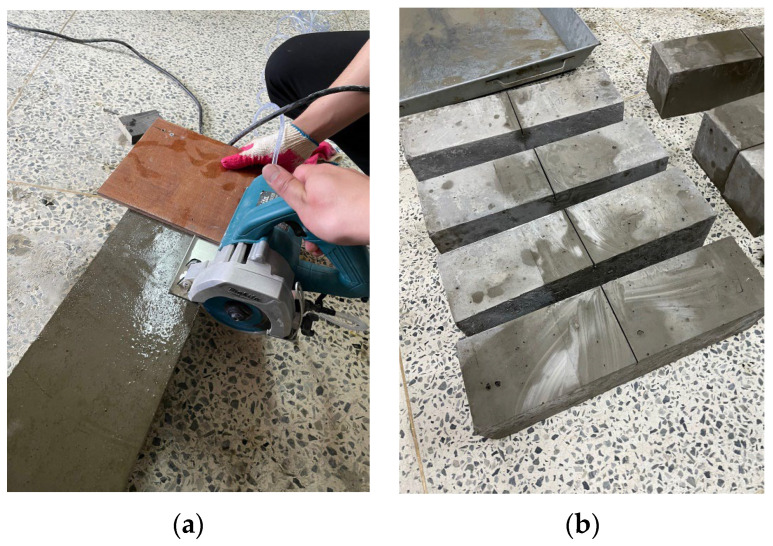
SFRC flexural test specimen: (**a**) Grinding specimen; (**b**) Specimens after grinding.

**Figure 3:**
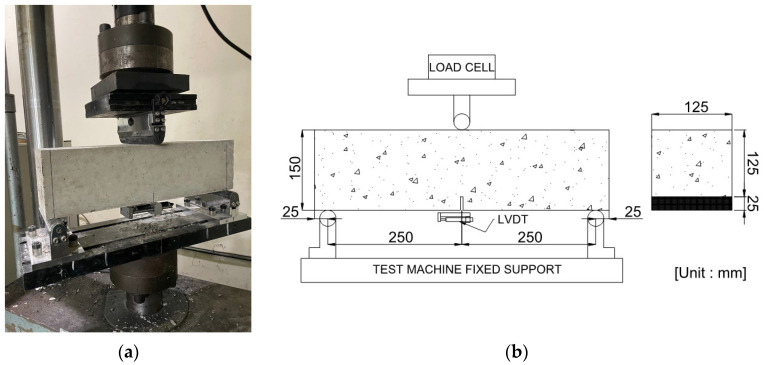
Test setup for an arched SFRC beam: (**a**) Test setup; (**b**) Schematic of test.

To evaluate the flexural properties of arched SFRC, specimens with a width, height, and length of 150 mm, 150 mm, and 550 mm, respectively, were prepared in accordance with EN 14651 [13]. The concrete was water cured for 28 days. Additionally, the temperature was maintained at 20 ± 2 °C. As shown in Figure 2, a notch of 25 ± 1 mm depth and 5 mm or less width was created at the center of each specimen. Hardware was attached to both sides of the notch to measure the crack mouth opening displacement (CMOD) through a linear variable differential transformer (LVDT). In the experiment, three-point loading was applied until fracture at a rate of 0.2 mm/min using a 200 kN actuator in accordance with EN 14651. Figure 3a shows the experimental setup and Figure 3b shows the schematic diagram of the experiment.

### 2.3. Residual Flexural Strength and Energy Absorption Capacity

In accordance with the EN code, to evaluate the flexural properties of arched SFRC, three-point loading was applied to the specimens until fracture. Based on the EN code, the flexural stress for each section was calculated using Equations (1) and (2), and the stress corresponding to the CMOD values of 0.5, 1.5, 2.5, and 3.5 m was determined.

(1) $$f_{R,J}=\frac{3F_{j}l}{2bh_{sp}^{2}},$$

where $f_{R,J}$: residual flexural stress (MPa) according to CMOD (j = 1,2,3,4), $F_{j}$: load (N) according to CMOD (j = 1,2,3,4), l: specimen length (mm), b: specimen width (mm), and $h_{sp}^{}$: distance between the end of the notch and the top of the specimen (mm).

The limit of proportionality (LOP) of the flexural strength of SFRC is given by Equation (2). The LOP flexural strength represents the maximum load within a CMOD value of 0.05 mm [13].

(2) $$f_{L}=\frac{3F_{L}l}{2bh_{sp}^{2}},$$

where $f_{L}$ is the maximum load (N) within a CMOD value of 0.05 mm.

According to RILEM TC 162-TDF [14], the energy absorption capacity can be calculated using the area under the load–displacement curve. In RILEM TC 162-TDF, areas with displacements of 0.7 and 2.7 mm are expressed as energy absorption capacity except for the triangular area at a displacement of 0.05 mm, which corresponds to FL. The CMOD displacement relationship was obtained using Equation (3), according to the EN codes. After converting CMOD to displacement using Equation (3), the load–displacement curve was drawn, and the energy absorption capacity was calculated.

(3) δ=0.85CMOD+0.04,

where δ: displacement (mm) and CMOD: crack mouth opening displacement (mm).

In Model Code 2010 [15], the serviceability limit state of fiber-reinforced concrete was expressed using the residual flexural strength at a CMOD value of 0.5 mm, and the ultimate limit state was expressed using the residual flexural strength at a CMOD value of 2.5 mm. Model Code 2010 also suggests that some or all of the tension bars in the concrete can be replaced if Equations (4) and (5) are satisfied in the ultimate limit state.

(4) $$f_{1}/f_{LOP}>0.4$$

(5) $$f_{3}/f_{1}>0.5$$

## 3. Flexural Behavior Measurement Results and Analysis for Arched SFRC

### 3.1. Results of Flexural Test

**Figure 4:**
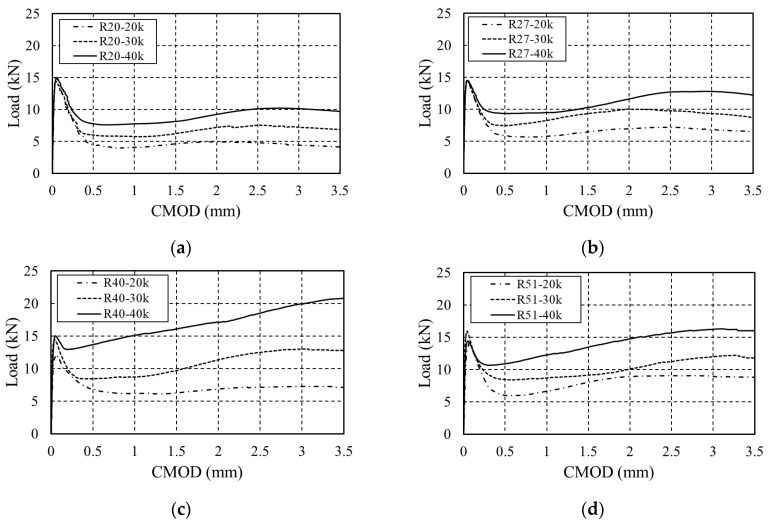
Load–CMOD curves of arched SFRC: (**a**) Arched_R20; (**b**) Arched_R27; (**c**) Arched_R40; (**d**) Arched_R51.

The load–CMOD curve of arched SFRC as a function of the steel fiber content is shown in Figure 4. The residual flexural stress for each section is summarized in Table 3. The behavior up to LOP showed no difference depending on the fiber geometry or content, possibly because the distribution of steel fibers before initial cracking had almost no effect on the mechanical properties of SFRC [7]. However, after LOP, the flexural strength increased with the content regardless of the fiber geometry, and ductile behavior was observed. When R20 was used, the flexural strength increased by 34.14% and 73.83% at a CMOD value of 0.5 mm (serviceability limit state) compared to 20 kg/m^3^, depending on the content. At a CMOD value of 2.5 mm (ultimate limit state), it increased by 57.38% and 111.06% compared to 20 kg/m^3^. For other fibers with different geometries, the residual flexural strength tended to increase by approximately 4.08–191.18% for each section, indicating that the bridging action of the fibers used in this study increased with increasing content regardless of their geometry.

**Figure 5:**
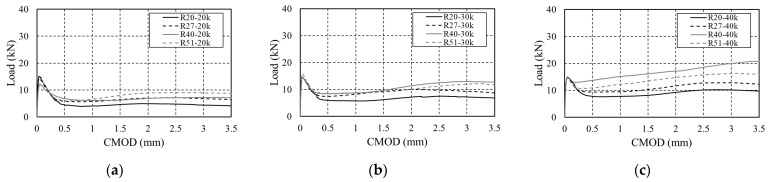
Load–CMOD curve by volume of steel fiber: (**a**) 20 kg/m^3^; (**b**) 30 kg/m^3^; (**c**) 40 kg/m^3^.

Figure 5 shows the load–CMOD curve according to the geometry of the steel fibers. At a fiber content of 20 kg/m^3^, R51 exhibited the highest residual stress, followed by R40, R27, and R20. However, at 30 and 40 kg/m^3^ fiber content, R40 showed the highest residual stress, followed by R51, R27, and R20. At a fiber content of 40 kg/m^3^, the residual stress at a CMOD value of 3.5 mm (ultimate limit state) increased by approximately 30–114.5% when each fiber was changed to R40, indicating that adding steel fiber 80 with 40 kg/m^3^ of R40 is effective when arched steel fibers are used in the 35 MPa concrete.

Table 4 summarizes the values required for replacing tension bars in concrete according to Equations (4) and (5) specified in Model Code 2010 [15]. It was found that the mixes used in this study can replace the tension bars in concrete, except in the case of adding 20 kg/m^3^ of R20, when the reinforcement is performed using arched steel fibers. The specimen to which R20 was added at a content of 20 kg/m^3^ met the F_3_/F_1_ value specified in the standard but could not reach the F_1_/F_LOP_ value. For the 35 MPa concrete used in the test, it was found that R20, with a relatively low aspect ratio and low curvature, can replace the tension bars when its content exceeds 30 kg/m^3^. Model Code classifies residual flexural strength after cracking into classes A, B, C, D, and E. Classes A and B were not observed when classification was performed based on the standard. The cases where 20 kg/m^3^ of R20 and R40 were added belonged to class C, and the cases where 30 and 40 kg/m^3^ of R20 were added belonged to class D. The remaining mixes belonged to class E.

**Figure 6:**
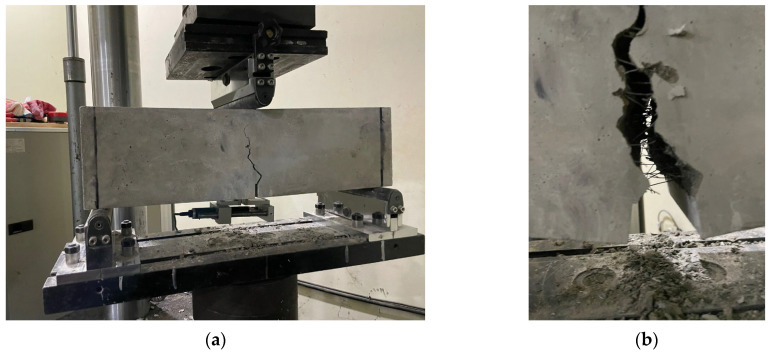
Cracking shape of arched SFRC: (**a**) Cracking shape; (**b**) Fiber’s bridging action.

Figure 6 shows the crack pattern of the R40-40k specimen after loading until fracture. The cracks propagated upward along the created notch in all specimens, as shown in Figure 6a. As shown in Figure 6b, the concrete fiber attachment effect, i.e., the bridging action of the fibers, was observed inside the crack. No fracture of the fibers was observed, indicating that the length of the fibers used in this study is not short enough to cause fracture and that they can provide sufficient pullout strength.

### 3.2. Energy Absorption Capacity

**Figure 7:**
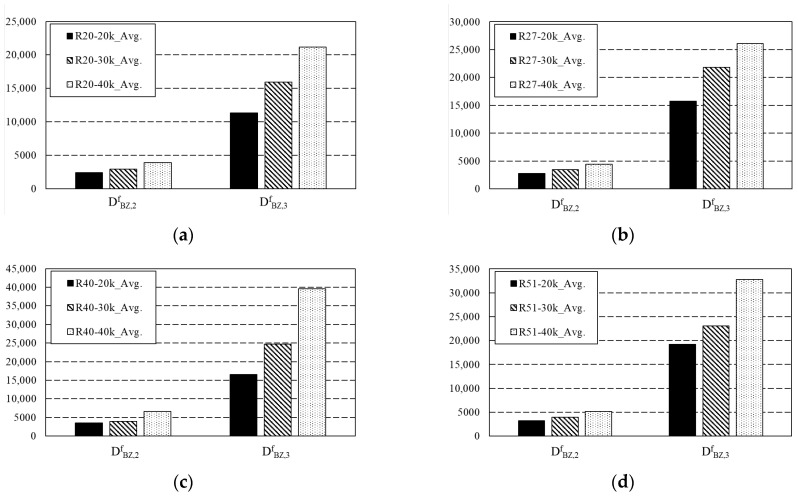
Energy absorption capacity of arched SFRC: (**a**) R20 arched SFRC; (**b**) R27 arched SFRC; (**c**) R40 arched SFRC; (**d**) R51 arched SFRC.

To evaluate the energy absorption capacity of arched SFRC, the experimental variables were compared and evaluated based on the RILEM TC 162-TDF standard [14]. Based on the standard, the triangular area at the value of F_L_ and the specified displacement was excluded from the total area to be obtained, and energy absorption capacity was expressed in joules (N·m). The standard specifies that the data must be excluded when the first cracking occurs outside the notch. In particular, D^f^_BZ,2_ and D^f^_BZ,3_ quantify the steel fibers energy absorption capacity. According to the standard, the area up to a displacement of 0.7 mm and up to 2.7 mm was determined as energy absorption capacity. The energy absorption capacity up to 0.7 mm was expressed as D^f^_BZ,2_ and that up to 2.7 mm as D^f^_BZ,3_, corresponding to the CMOD values of the serviceability limit state and ultimate limit state given in the Model Code, respectively. Figure 7 shows the energy absorption capacity according to the content of arched steel fibers, while Table 5 summarizes the values of energy absorption capacity values shown in Figure 7. For the specimen shown in Figure 7 and Table 5, labeled R20-20k, R20 represents the curvature of the fiber and 20k indicates a content of 20 kg/m^3^. Regardless of the geometry of the fibers, the energy absorption capacity increased with increasing fiber content at both 0.7 and 2.7 mm sections. D^f^_BZ,2_ at 30 kg/m^3^ compared to 20 kg/m^3^ increased by approximately 16.15–23.81%. The increment of concrete that used R40 was relatively low, while the increment of concrete that used R27 was relatively high. D^f^_BZ,2_ at 40 kg/m^3^ compared to 20 kg/m^3^ increased by approximately 57.64–89.31%. When R40 was used, the increment was relatively high. Among the mixes used in this study, the addition of 40 kg/m^3^ of R40 was judged to be the most effective in the serviceability limit state.

Regarding the increment up to 2.7 mm, the specimen using R40 exhibited the highest values of 48.87 and 138.88% compared to the specimen with a content of 20 kg/m^3^. Compared to a content of 20 kg/m^3^, the energy absorption capacity increased by 40.9 and 86.96% for R20-reinforced concrete, 38.46 and 65.40% for R27-reinforced concrete, and 20.17 and 70.64% for R51-reinforced concrete. The highest increment in energy absorption capacity was observed when 40 kg/m^3^ of R40 was added. Similar to the flexural test results, the R40-40k mix was considered efficient for the 35 MPa concrete used in this study.

**Figure 8:**
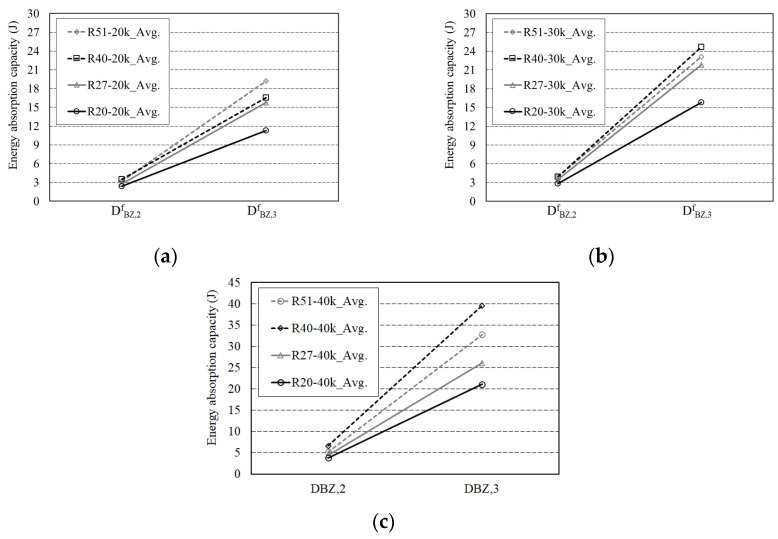
Energy absorption capacity of arched SFRC: (**a**) 20 kg/m^3^ steel fiber; (**b**) 30 kg/m^3^ steel fiber; (**c**) 40 kg/m^3^ steel fiber.

Figure 8 shows the energy absorption capacity according to the geometry of the steel fibers. At a steel fiber content of 20 kg/m^3^, D^f^_BZ,2_ increased in the order of R20, R27, R40, and R51 according to the curvature of the fiber as shown in Figure 8a. Compared to R20, R27, R40, and R51 exhibited 16.6, 44.97, and 32.52% higher energy absorption capacity, respectively, as shown in Figure 8b. When R40 was changed to R51, the curvature of the fiber increased while its energy absorption capacity decreased by 8.59% as shown in Figure 8c. At a steel fiber content of 20 kg/m^3^, D^f^_BZ,3_ increased in the order of R20, R27, R40, and R51 according to the curvature of the fiber, indicating that the energy absorption performance in the ultimate limit state improved with increasing curvature of the steel fiber at a steel fiber content of 20 kg/m^3^. At 30 and 40 kg/m^3^ steel fiber content, D^f^_BZ,2_ and D^f^_BZ,3_ were higher, in the order of R20, R27, R51, and R40. At a steel fiber content of 40 kg/m^3^, the energy absorption capacity of R40 was approximately 72.68–87.31% higher than that of R20. In terms of energy absorption capacity, the R40-40k mixture is deemed the most effective among the mixtures used in this study.

## 4. Results of Residual Stress Measurement and Analysis for Arched SFRC

To compare the residual stress of arched SFRC by section, the residual stress by section was divided by the square root of the concrete strength based on the method presented by Carrillo et al. [16]. In addition, the flexural test results of previous studies [7,8,16,17] and the test results of this study were standardized and compared using Equation (6) to determine the proper distribution of residual stress by section.

RI = Vf × lf/df,(6)

where RI is the reinforcement index, representing the fiber content and aspect ratio which determine the mechanical properties of fiber-reinforced concrete and Vf is the fiber content. lf/df is the aspect ratio of the fibers. As the value of LOP, which is the initial crack, is not affected by the fibers, the average value was calculated and compared. In addition, the average value of the residual flexural strength per section was divided by the square root of the concrete compressive strength to standardize and compare the flexural test results in each study.

**Figure 9:**
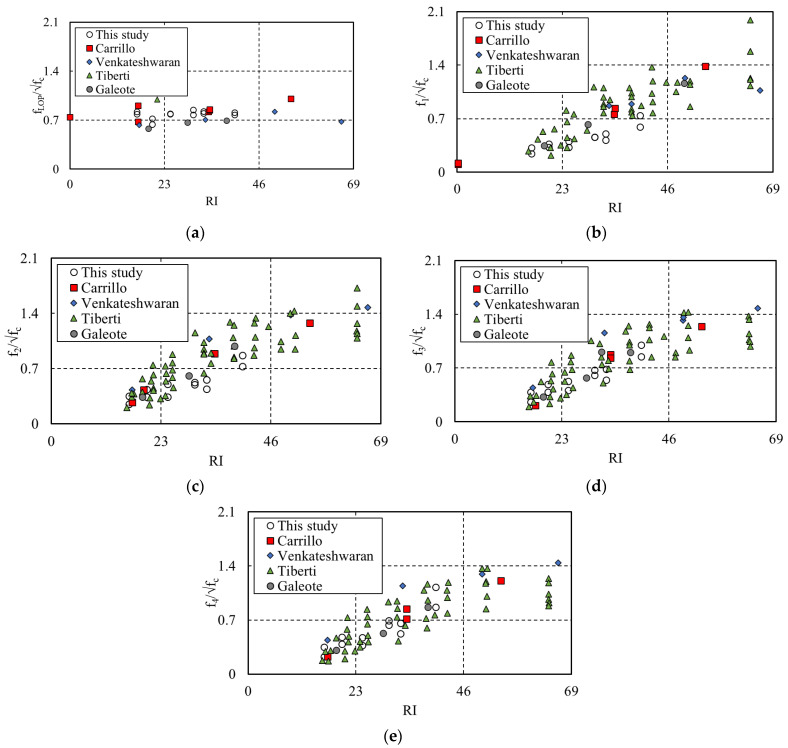
Different studies test results of SFRC beam flexural tests: (**a**) Results of F_LOP_; (**b**) Results of F_1_; (**c**) Results of F_2_; (**d**) Results of F_3_; (**e**) Results of F_4_.

The test results of previous studies [7,8,16,17] and those of this study were divided by the residual stress per section, with the results shown in Figure 9. Considering the F_LOP_ results shown in Figure 9a, the average values of the results of previous studies were 0.83, 0.71, 0.59, and 0.83 in the order of the authors listed above. The average F_LOP_ result of this study was 0.8, differing from the results of previous studies by 3.75–26.25%. Carrilo et al. and Caleote et al. [16,17] differed by 3.75% from the average value (0.83). Furthermore, Venkateshwaran et al. [7] exhibited a difference of 11.2% compared to the average value (0.71). The average value (0.59) of Tiberti et al. [8] presented the largest difference (26.25%) from the result of this study. The difference in the F_LOP_ result is related to the geometry of the aggregate or the air content and admixture, which affected the mechanical properties of the concrete matrix subjected to tension even though the distribution of steel fibers did not affect the initial cracking.

**Figure 10:**
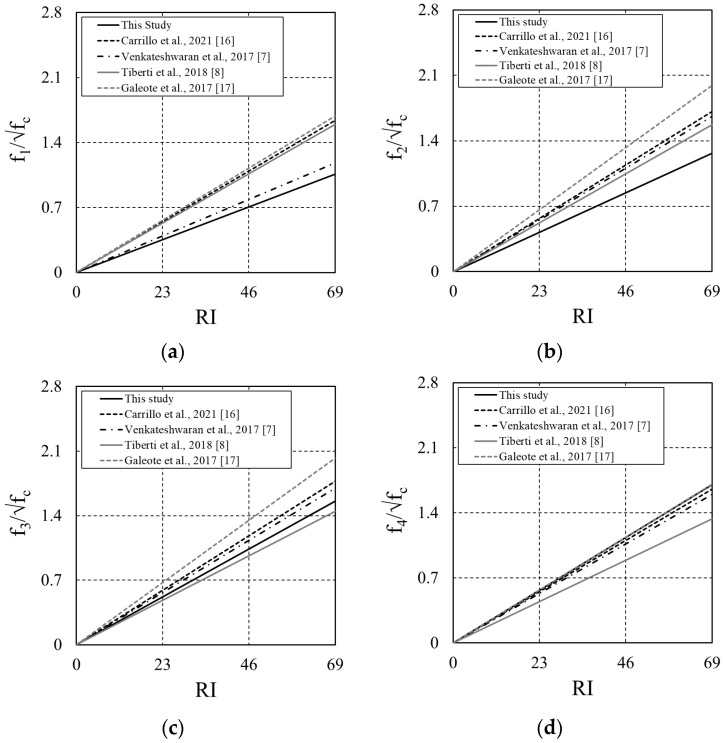
Trend line between this study and other studies: (**a**) Results of F_1_; (**b**) Results of F_2_; (**c**) Results of F_3_; (**d**) Results of F_4_.

Trend lines corresponding to the test results shown in Figure 9 were compared. The results are shown in Figure 10. R^2^ values for residual stress by section were 0.84, 0.78, 0.86, and 0.82 for F_1,_ F_2,_ F_3,_ and F_4_, respectively. The residual flexural stress increased with increasing RI, indicating similar behavior to the results of previous studies. The behavior observed in this study differed from the research results of Tiberti et al. [8], where high residual stresses in the early sections (F_1_ and F_2_) and relatively low values in sections F_3_ and F_4_ were observed.

## 5. Conclusions

In this study, a flexural test of 35 MPa concrete for different geometries (65 and 80) and content (20, 30, and 40 kg/m^3^) of arched steel fibers were conducted according to EN 14651 to determine the flexural properties of arched steel fiber-reinforced concrete (arched SFRC).

1. Arched SFRC showed no difference in the residual flexural stress of the LOP section depending on the fiber geometry and content. The residual flexural stress after cracking increased by approximately 34.14–73.83% in the serviceability limit state and 57.38–111.06% in the ultimate limit state as the fiber content increased. In addition, the residual flexural stress increased by approximately 30–114.5% depending on the fiber geometry. The specimen mixed with the R40 fiber exhibited the highest residual stress.
2. When classifying the SFRC specimens according to the Model Code 2010, R20-20k and R40-20k were found to belong to class C. Class D included R20-30k and 40k and R27-20k, while class E included R27-30k and 40k, R40-30k and 40k, and R51-20k, 30k, and 40k. The specimens with high fiber curvatures and fiber content tended to belong to high classes.
3. The energy absorption capacity of arched SFRC increased with increasing curvature and fiber content of fibers. The R40-40k specimen showed the highest energy absorption capacity. The capacity increased by approximately 48.87–138.88% as the content of the R40 fiber increased.
4. The comparison of the experimental results of this study to those of previous studies that implemented hooked steel fiber, i.e., Carrillo et al., Venkateshwaran et al., Tiberti et al., Galeote et al. [7,8,16,17] confirmed that the arched steel fibers used exhibited comparable performance to the hooked steel fibers. In the proportional linearity section, the difference between hooked and arched steel fibers was approximately 3.75%. In addition, the R^2^ value of the trend lines confirmed the reliability of the experimental results; the R^2^ value, ranged from 0.78–0.86.

## Figures and Tables

**Table 1:** Mix proportion of 35 MPa concrete.

W/B (%)	S/a (%)	Unit Weight (kg/m^3^)								AD, B *%
		W	C	FA	BS	S	CS	G	S. F	
36.4	55.1	160	286	66	88	662	283	770	20	1.4
									30	
									40	

W: water, C: cement, FA: fly ash, BS: ground granulated blast furnace slag, S: sand, CS: crushed sand, G: coarse aggregate, S. F.: steel fiber, and AD, B *%: superplasticizer.

**Table 2:** Description of the arched steel fiber-reinforced concrete flexural test specimens.

Steel Fiber Type	Length	Content (kg/m^3^)	Tensile Strength of Steel Fibers (MPa)	Compressive Strength of Concrete (MPa)	Number of Specimens
Steel fiber 65	R20	20	1250	35	4
		30			4
		40			4
	R27	20			4
		30			4
		40			4
Steel fiber 80	R40	20	1100		4
		30			4
		40			4
	R51	20			4
		30			4
		40			4

**Table 3:** Test results of arched SFRC.

Fiber Geometry	Fiber Content (kg/m^3^)	F_LOP_ (MPa)	F_1_ (MPa)	F_2_ (MPa)	F_3_ (MPa)	F_4_ (MPa)
R20	20	4.84	1.43	1.48	1.53	1.33
	30	4.64	1.92	1.99	2.41	2.20
	40	4.88	2.49	2.60	3.23	3.10
R27	20	4.66	1.89	2.08	2.30	2.07
	30	4.68	2.39	2.99	3.12	2.79
	40	4.71	2.99	3.29	4.07	3.91
R40	20	3.79	2.17	2.01	2.28	2.29
	30	4.59	2.70	3.10	3.99	4.09
	40	4.79	4.38	5.15	5.93	6.66
R51	20	4.28	1.92	2.57	2.90	2.82
	30	5.02	2.71	2.92	3.59	3.77
	40	4.61	3.49	4.31	5.02	5.12

**Table 4:** SFRC flexural relationships.

Description	F_1_/F_LOP_	F_3_/F_1_	Class
R20-20k	0.30	1.07	C
R20-30k	0.41	1.25	D
R20-40k	0.51	1.22	D
R27-20k	0.41	1.22	D
R27-30k	0.51	1.31	E
R27-40k	0.64	1.36	E
R40-20k	0.57	1.05	C
R40-30k	0.59	1.48	E
R40-40k	0.91	1.35	E
R51-20k	0.45	1.51	E
R51-30k	0.54	1.33	E
R51-40k	0.76	1.44	E

**Table 5:** Energy absorption capacity of arched SFRC.

Description	D^f^_BZ,2_	D^f^_BZ,3_
R20-20k_Avg.	2.41	11.31
R20-30k_Avg.	2.80	15.81
R20-40k_Avg.	3.83	21.14
R27-20k_Avg.	2.81	15.78
R27-30k_Avg.	3.48	21.85
R27-40k_Avg.	4.43	26.10
R40-20k_Avg.	3.49	16.57
R40-30k_Avg.	3.92	24.69
R40-40k_Avg.	6.61	39.59
R51-20k_Avg.	3.19	19.22
R51-30k_Avg.	3.91	23.10
R51-40k_Avg.	5.17	32.80

## Data Availability

The data presented in this study are available on request from the corresponding author.
